# A systematic review of economic evaluations of population-based sodium reduction interventions

**DOI:** 10.1371/journal.pone.0173600

**Published:** 2017-03-29

**Authors:** Silvia F. Hope, Jacqui Webster, Kathy Trieu, Arti Pillay, Merina Ieremia, Colin Bell, Wendy Snowdon, Bruce Neal, Marj Moodie

**Affiliations:** 1 Deakin Health Economics, Centre for Population Health Research, Faculty of Health, Deakin University, Burwood, Victoria, Australia; 2 The George Institute for Global Health, Sydney, Australia; 3 Pacific Research Centre for Prevention of Obesity and Non Communicable Diseases (C-POND)/ Fiji National University, Suva, Fiji; 4 Ministry of Health, Apia, Samoa; 5 Global Obesity Centre, Faculty of Health, Deakin University, Melbourne, Australia; 6 The Charles Perkins Centre, University of Sydney, Sydney, Australia; 7 Division of Epidemiology and Biostatistics, Imperial College, London, United Kingdom; St. Michael's Hospital, CANADA

## Abstract

**Objective:**

To summarise evidence describing the cost-effectiveness of population-based interventions targeting sodium reduction.

**Methods:**

A systematic search of published and grey literature databases and websites was conducted using specified key words. Characteristics of identified economic evaluations were recorded, and included studies were appraised for reporting quality using the Consolidated Health Economic Evaluation Reporting Standards (CHEERS) checklist.

**Results:**

Twenty studies met the study inclusion criteria and received a full paper review. Fourteen studies were identified as full economic evaluations in that they included both costs and benefits associated with an intervention measured against a comparator. Most studies were modelling exercises based on scenarios for achieving salt reduction and assumed effects on health outcomes. All 14 studies concluded that their specified intervention(s) targeting reductions in population sodium consumption were cost-effective, and in the majority of cases, were cost saving. Just over half the studies (8/14) were assessed as being of ‘excellent’ reporting quality, five studies fell into the ‘very good’ quality category and one into the ‘good’ category. All of the identified evaluations were based on modelling, whereby inputs for all the key parameters including the effect size were either drawn from published datasets, existing literature or based on expert advice.

**Conclusion:**

Despite a clear increase in evaluations of salt reduction programs in recent years, this review identified relatively few economic evaluations of population salt reduction interventions. None of the studies were based on actual implementation of intervention(s) and the associated collection of new empirical data. The studies universally showed that population-based salt reduction strategies are likely to be cost effective or cost saving. However, given the reliance on modelling, there is a need for the effectiveness of new interventions to be evaluated in the field using strong study designs and parallel economic evaluations.

## Background

As the non-communicable diseases (NCD) crisis becomes an urgent race against time [1], it is critical to understand the effectiveness of interventions designed to lower the risk factors associated with cardiovascular disease (CVD), which is now the leading cause of deaths globally [2]. Recent data highlight blood pressure as a leading risk to health [3], and one of the main causes of elevated blood pressure is excess dietary sodium intake [4,5].

Excess dietary sodium intake is likely to be responsible for about half of the disease burden ascribed to high blood pressure [6] making sodium a major contributor to mortality from CVD [7]. As a result, interventions targeting the reduction of population-wide sodium intake are increasingly being prioritised [8]. New Guidelines issued by the WHO in 2012 recommend that adults should consume less than 2000mg of sodium or 5 grams of salt per day [9]. This is significantly lower than the average intake in many countries such Samoa which averages 7.09 grams [10], Australia around 8 grams [11] and the United States 8.5 grams [12] per day. Recent estimations from the Global Burden of Disease study suggest that global salt intake is around 10grams/day [13]. For many countries, reaching the sodium guideline of 5 grams per day would require a 50% reduction in daily salt intake from current levels.

There is compelling evidence that a reduction in sodium intake significantly reduces resting systolic blood pressure [14] and is therefore likely to reduce the risk of a CVD event [15]. A high intake of sodium increases blood pressure levels with age, greatly increasing the risk of cardiovascular disease and contributing to nearly half the disease burden attributed to high blood pressure [16]. Evidence from epidemiology and from high quality analysis of randomized clinical trials shows a direct relationship between blood pressure and cardiovascular diseases [17–21]. There is also increasing evidence that population interventions to reduce salt are effective in reducing blood pressure [18,22]. Further evidence from a meta-analysis of randomized salt reduction trials estimated that a reduction in salt intake of 6 g/day would reduce the prevalence of strokes by 24% and coronary heart disease by 18% [15].

A range of interventions has been developed and implemented in efforts to reduce sodium consumption with the choice of salt reduction strategy depending upon the source of sodium in the diet [23]. In developed countries, the majority of sodium comes from processed foods such as bread, processed meat, cheese and fast food, whereas in developing countries, a greater proportion typically derives from salt added during cooking or at the table [15]. The main interventions for sodium reduction include product reformulation (both voluntary and mandatory), health promotion campaigns, mandatory labeling of salt content on pre-packaged food, and taxation or other incentives to encourage the food industry to moderate the level of salt in processed foods [23]. Sodium reduction interventions are commonly shown to be highly effective in reducing sodium intake at a population level. A recent evaluation of the salt reduction initiative in the United Kingdom of Great Britain and the Northern Ireland demonstrated a significant reduction in average intakes from 9.5 grams per day in 2000 to 8.1 grams per day in 2011 [24]. The initiatives consisted of an awareness campaign shown on TV through a series of adverts along with a series of partnerships with institutions running programs such as peer education and social cooking classes. The strategy also involved working with the food industry to encourage product reformulation.

Interventions that reduce sodium intake have been shown to be one of the most cost-effective measures to improve public health worldwide [25]. These interventions generally target whole populations and seek to reduce exposure to dietary sodium [26]. It is estimated that a 15% reduction in population-wide sodium consumption would avert up to 8·5 million deaths in 23 high-burden countries over 10 years [6].

Whilst numerous studies explore the effectiveness of sodium reduction interventions on salt intake through urine collections, effect on blood pressure or cardiovascular disease outcomes [14,19,27], decision makers are also interested in which interventions deliver value-for-money in the context of limited health care resources. Economic evaluations are extremely valuable in decision making as they enable the best course of action to be identified based on the evidence available by systematically analyzing the costs and benefits associated with an intervention and assessing its value for money [28]. Whilst there is broad agreement that sodium reduction strategies are cost-effective, there are many different evaluation approaches and perspectives used, and the completed evaluations vary in quality.

The objective of this paper was to conduct a systematic review of the literature to identify economic evaluation studies of interventions targeting sodium reduction and summarise evidence about their cost-effectiveness.

### Important definitions

A table of important definitions has been compiled below in Table 1, defining important terms used throughout this paper.

**Table 1:** Important definitions.

Term	Definition
Sodium	A mineral, and one of the chemical elements found in salt. Salt (sodium chloride) is made up of 40% sodium and 60% chloride. One teaspoon of table salt contains 2,325 mg of sodium [29].
Cost-effectiveness analysis (CEA)	An evaluation in which the effects of an intervention (and its comparators) are measured in identical units of outcome (e.g. mortality, myocardial infarctions) and alternative interventions are compared in terms of ‘cost per unit of effect’ [30].
Cost-utility analysis (CUA)	When alternative interventions produce different levels of effect in terms of both quantity and quality of life (or different effects), the effects may be expressed in utilities. Utilities are measures which comprise both length of life and subjective levels of well-being. The best known utility measure is the quality-adjusted life year, or QALY. Alternative interventions are compared in terms of cost per unit of utility gained (e.g. cost per QALY) [30].
Cost-benefit analysis (CBA)	When both resource inputs and effects of alternative interventions are expressed in monetary units, so that they compare directly and across programs within the healthcare system, or with programs outside health care (e.g. healthcare intervention vs. criminal justice intervention) [30].
Incremental cost-effectiveness ratio (ICER)	Entails determination of the incremental cost of an additional unit of health benefit thereby enabling different interventions to be ranked in terms of their economic credentials. The ICER is calculated by difference in cost between two possible interventions, divided by the difference in their effect [30].
Economic Perspective	A viewpoint that envisions individuals and institutions making rational decisions by comparing the marginal benefits and marginal costs associated with their actions [31].

## Methods

### Search strategy

#### Databases searched

Literature on economic evaluations of sodium reduction interventions published between 1980 and 2015 were identified from a search of journal databases, grey literature and other articles identified by experts in the field. During January 2015, the published literature was searched using the following search engines which comprise the main health databases: Pubmed, Embase, EBSCO Host, OVID and Google Scholar. This review explores the existing literature on both economic evaluations of sodium reduction interventions actually implemented in the field and involving new empirical data collection as well as desk-based modelled simulation studies.

A search of grey literature was also undertaken using the same search terms in order to find information that may only have been published in government reports or discussion papers. The search was undertaken using Google, Open Grey, the World Health Organization database and website and the World Bank website. The reference lists of extracted articles were also searched for any additional studies.

#### Search terms

Each database was searched using the following key words: “Economic Evaluat*”, “Cost Effect*”, “Cost Benefit”, “Cost Utility”, “Cost Analyses” and “Intervention*”. Each search term was combined with the key words “Sodium OR Salt” and “Reduc*”.

#### Study inclusion criteria

To be included, a study had to comply with all of the following criteria:

1. Be an intervention or simulation study that targeted the reduction of sodium intake at a population level (i.e. targeting populations rather than individuals). Both prospective and retrospective studies were included.
2. Presented the findings of full economic evaluations which explore both costs and benefits in relation to a comparator. A full economic evaluation was defined as the comparative analysis of alternative courses of action in terms of both costs (resource use) and consequences (outcomes, effects) [30]. Full economic evaluations include studies utilising CBA, CEA or CUA. Partial economic analyses, which focused solely on costs and resource used, or which did not entail a comparator, were excluded.
3. Published from 1980 to December 2015.
4. Reported in English.

The systematic review was conducted by SH following the Preferred Reporting Items for Systematic Reviews and Meta-Analyses (PRISMA) guidelines [32]. The results were identified by title, then screened by abstract, followed by a full text assessment for eligibility.

#### Analyses

Key characteristics of the economic evaluation of each of the identified sodium reduction studies were extracted into a spreadsheet including the economic evaluation study design, year and country of study, setting, sample size, time horizon, study perspective, study comparator, intervention(s) analyzed, the methods or models used to conduct the economic evaluation, costs included, the primary outcome measure and the main results and conclusions of the study.

The reporting quality of the identified studies was measured against the Consolidated Health Economic Evaluation Reporting Standards (CHEERS) checklist for assessing economic evaluations [33,34]. The 24 item checklist is designed to improve reporting of economic evaluations. Each of the included articles were assessed for reporting quality independently by two reviewers (SH, MM) against the criteria to calculate a score out of 24 (or the number of applicable items). Each item on the checklist was assigned one point, but half points were awarded where the article partially filled the criteria (e.g. provided no explanation for choice of discount rate or choice of model). The two reviewers (SH and MM) discussed any differences in criteria ratings in order to reach consensus. A percentage score for each study was then calculated. In the absence of a broadly accepted method for reporting quality appraisal, categories were set based on methods from other literature [35–37]—a study was deemed to be of excellent reporting quality if it scored 85% or higher, 70-<85% very good quality, 55-<70% good quality and studies scoring below 55% were classified as poor quality.

## Results

### Search results

From the initial search, a total of 3647 potentially relevant publications were identified. Some 924 duplicates were removed. Of the remaining 2723 titles, 2639 were found to be not relevant based on the title key words. A review of the abstracts of the remaining 84 articles identified a total of 25 potentially relevant studies. After a partial review of the full article, a further 10 papers were excluded as they did not meet the selection criteria (seven did not present findings of an economic evaluation, two were not available in English, and one was a conference abstract).

In addition to the fifteen articles identified from the database search, an additional five articles were identified through either the grey literature search or referral from persons working in the field. So in total, 20 studies were identified that met the study inclusion criteria and were subjected to a full paper review [22,38–56].

**Fig 1:**
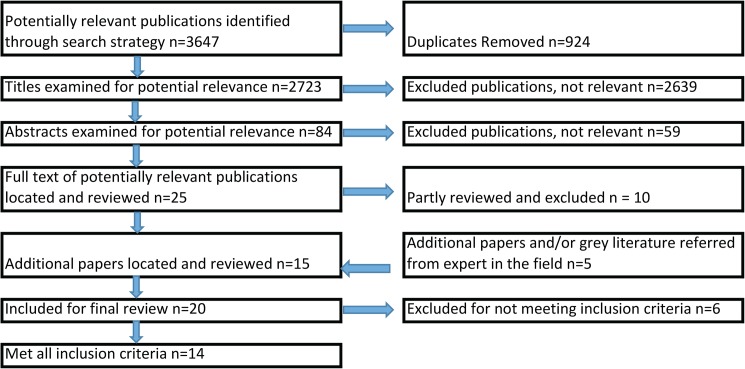
Flow diagram of selection process, according to PRISMA Guidelines [32].

Five of the twenty studies [22,38–41], whilst purporting to be cost-effectiveness analyses, did not actually specify an intervention. Another [56] was excluded as it was a protocol. Fourteen articles [42–55] were full economic evaluations in that they included both costs and benefits associated with an intervention measured against a comparator. A flow diagram of the selection process, according to the PRISMA Guidelines is shown below in Fig 1 [32].

The majority of studies (11/14) have been published in the past five years (2010 or later), with the other three studies being in the decade 2000–2009. This is a reflection of both the relative newness of attention being focused on sodium reduction interventions as a measure to curb hypertension, and of the infancy of the exposure of such interventions to economic evaluation.

The 14 papers outlined in Table 2 [42–55] were full economic evaluations in that they included both costs and benefits associated with a defined intervention measured against a comparator. The characteristics of these papers are summarised in Table 3 and explained in the following section.

**Table 2:** Study results–full economic evaluations.

Authors, Country, Year	Title	Study design, study perspective	Target population	Intervention, comparator	Time horizon, discount rate	Methods or Model	Costs (sources)	Reporting of costs	Economic Benefits	Conclusions	Measure of health benefit	Cost effective/cost saving
Selmer et al (42)., Norway, 2000	Cost and health consequences of reducing the population intake of salt.	CUA Societal	1995 Norwegian Population aged 40 and over	Health promotion; reformulation, labelling; taxes on salty food/subsidies of products with less salt. Comparator: current practice	Intervention duration: 25 years. Timeframe of costs and consequences evaluated: 25 years. Discount rate 5%	Dynamic simulation modelling using a Markov model.	Information campaigns and new food industry products (expert opinion); welfare costs associated with taxation/ subsidy (based on price elasticity of such food); cost savings from avoided events (DRG data).	Measured in 1997 Kr ($1 = Kr7).	Estimated net savings of program over 25 years were $270 million.	Population interventions to reduce salt intake are likely to improve the population's health and save costs to society. Cost saving	Risk of myocardial infarction and stroke mortality. Assumed the effects appear gradually over five years from the onset of intervention.	Cost Saving (assumed that the cost savings from one avoided non-fatal stroke would be $14 286).
Wilcox et al (43)., Syria, 2014	Cost-effectiveness analysis of salt reduction policies to reduce coronary heart disease in Syria, 2010–2020.	CUA perspective not specified	Syrian adults aged 25 and older	Health promotion (HP); mandatory labelling (L); mandatory reformulation (R); combination of all three policies. Comparator: current practice	Costs and benefits measured over 10 years (2010–2020). Discount rate 3%	Modelled policy analyses using IMPACT Coronary Heart Disease model	Net cost of each policy = total policy cost—health care savings related to CHD. Costs of Health promotion campaign and of policy development and enforcement (historical data and expert opinion); costs of product relabeling and reformulation (interviews with manufacturers); treatment costs (Ministry of Health)	Reported in 2010 local currency values, then converted to international dollars using purchasing power parity exchange rates. All future costs inflated by country-specific inflation rate of 4.4% and then discounted by 3%.	HP, L, and R + HP + L = cost-saving. Remaining policies cost-effective (CERs: R = $5,453 PPP/LYG; R + HP = $2,201 PPP/LYG; R + L = $2,125 PPP/LYG). R + HP + L provided the largest benefit with net savings using the best and maximum estimates, while R + L cost-effective with the lowest marginal cost using the minimum estimates.	All policies were cost-saving or cost-effective. The combination of reformulation, labelling and a comprehensive policy involving all three approaches was the most promising strategy.	LYGs based on literature. intervention effects were based on literature and assumed to be maintained over 10 years	Cost effective and cost saving (Policies with ratios\$13,000 PPP/ LYG were deemed as very cost effective, and those with ratios between $13,000 and $38,997 PPP/LYG were deemed as cost effective)
Mason et al (44)., Tunisia, Syria, Palestine, Turkey, 2014	A Cost Effectiveness Analyses of Salt Reduction Policies to Reduce Coronary Heart Disease in Four Eastern Mediterranean Countries	CUA Societal	Full population—Tunisia, Syria, Palestine, Turkey	Health promotion; labeling; mandatory reformulation; combination of all three policies. Comparator: Current practice (no policy)	Timeframe of costs and consequences evaluated: 10 years. Discount rate 3%	Modelled policy analyses using IMPACT Coronary Heart Disease model and country specific data	The costs of each policy were estimated using evidence from comparable policies and expert opinion including public sector costs and costs to the food industry. Health care costs associated with CHDs were estimated using standardized unit costs.	All costs were calculated using 2010 PPP exchange rates.	In all four countries, most policies cost saving. The combination of all three policies resulted in estimated cost savings of $235,000,000 and 6455 LYG in Tunisia; $39,000,000 and 31674 LYG in Syria; $6,000,000 and 2682 LYG in Palestine and $1,3000,000,000 and 378439 LYG in Turkey.	A comprehensive strategy of health education and food industry actions to label and reduce salt content would save both money and lives	LYG based on literature, intervention effects were based on literature and assumed to be maintained over 10 years.	Cost saving
Collins et al (45)., England, 2014	An Economic Evaluation of Salt Reduction Policies to Reduce Coronary Heart Disease in England: A Policy Modeling Study	CUA Societal	England population over 16 years	Four interventions: 1. Change4Life Health promotion campaign; 2. Front-of-pack traffic light labeling to show salt content; 3. Voluntary reformulation; 4. Mandatory reformulation. Comparator: Current Practice	Timeframe of costs and consequences evaluated: 10 years. Discount rate 3.5%	Modelled policy analyses using IMPACT Coronary Heart Disease model	Policy costs (published evidence). Health promotion campaign (Dept of Health); product labelling and reformulation (FSA Impact Assessment 2009 and British Retail Consortium); policy monitoring (FSA). Health care costs (Dept of Health reference costs 2010–2011; Unit Costs of Health and Social Care 2011).	Costs were reported in British Pounds based on a 2010 reference year.	Change4Life 984 LFY (606–1,424); labeling 984 LFY (606–1,424); voluntary reformulation 4,902 LFY (3,024–7,085); mandatory reformulation (best case) 9,758 LFY (6,030–14,080); (worst case) 9,758 LFY (6,030–14,080)	Population health interventions that effectively reduce dietary salt intake in the English population could substantially decrease health care expenditure and CVD burden. Mandatory reformulation of processed foods achieve the biggest reductions in dietary salt intake and the largest savings	Mortality and LYG based on literature, intervention effects were based on literature and assumed to be maintained over 10 years.	Cost saving
Cobiac et al (46)., Australia, 2010	Cost-effectiveness of interventions to reduce dietary salt intake.	CUA Health sector	Australian population in 2003	Four interventions: 1. Tick program and voluntary reformulation; 2. legislation and mandatory reformulation; 3.dietary advice for people at increased CVD risk (SBP>115 mm Hg; 4. dietary advice for persons at high CVD risk (SBP>140mm Hg). Comparator: current practice	Timeframe of costs and consequences evaluated: lifetime. Discount rate: 3%	Proportional multistate life-table modelling of cardiovascular disease and health sector cost outcomes	Food industry program costs (number of participating products and average annual cost per product); excludes food manufacturer costs associated with adding less salt. Costs of changing legislation (WHO estimates). Dietary advice (assumed costs of recruitment and dietitian delivery accrue to government). Patient time and travel costs included. Health care costs (Aust Institute of Health and Welfare Costs and Disease Impacts study).	All costs are adjusted to Australian dollars for the year 2003.	A total of 610 000 DALYs averted (480 000; 740 000) over cohort's lifetime if everyone reduced their salt intake to recommended limits. Making tick limits mandatory for all bread, margarine and cereal products could avert 18% of the disease burden, which is 20 times the health gain achieved with the voluntary approach.	Programs to encourage the food industry to reduce salt in processed foods are highly recommended for improving population health and reducing health sector spending in the long term, but regulatory action from government may be needed to achieve the potential of significant improvements in population health.	DALYs averted based on literature, Assumed prevention effects are based on literature and assumed to be fully realised within one year and then maintained.	Cost effective (based on an Australian cost-effectiveness threshold of A$50 000 per DALY
Murray CJ et al (47)., USA, 2005	Effectiveness and costs of interventions to lower systolic blood pressure and cholesterol: a global and regional analysis on reduction of cardiovascular-disease risk.	CUA Societal	14 epidemiological sub regions of the world	17 non-personal (health education through mass media, and either legislation or voluntary agreements on labelling and salt content) and personal interventions (treatment of persons with high cholesterol and/or high SBP and those at high risk of a CVD event in next 10 years. Comparator: Current practice	Timeframe of costs and consequences evaluated: 100 years (lifetime). Discount rate: 3%	Modelled analysis using PopMod. Effect sizes were derived from systematic reviews or meta-analyses, and the effect on health outcomes projected over time for populations with differing age, sex, and epidemiological profiles. Incidence data from estimates of burden of disease were used in a four-state longitudinal population model to calculate disability-adjusted life years (DALYs) averted and patients treated.	Costs (program and patient level costs)—based on previous publications, and local expert opinion in each region.	Costs reported in local currencies are converted to international dollars using purchasing power parities. Base year = 2008.	63 Million DALYs per year averted world wide	Non-personal health interventions to reduce blood pressure and cholesterol very cost effective, but personal health service strategies have greater potential to reduce disease burden.	DALYs averted based on literature, intervention effects were based on literature and assumed to be maintained.	Cost effective (based on interventions that gain each year of healthy life (eg, DALY averted) at a cost less than GDP per head are defined as very cost effective; those averting each DALY at a cost between one and three times GDP per head are cost effective; and the remainder are not cost-effective.
Cobiac et al (48)., Australia, 2012	Which interventions offer best value for money in primary prevention of cardiovascular disease?	CUA Health sector	Australian men and women, aged 35 to 84 years, who have never experienced a CVD event (angina, myocardial infarction, and stroke).	One salt intervention amongst 9 targeting CVD—mandatory reduction of salt in manufacture of breads, margarines and cereals. Comparator: current practice	Timeframe of costs and consequences evaluated: lifetime. Discount rate: 3%	Discrete time Markov model used to simulate CVD outcomes and cost impacts over cohort's lifetime	Costs of voluntary reformulation (based on % products participating in the current Heart Foundation program and the annual fee per product). Costs of legislative changes and enforcement (WHO unit costs)	Costs adjusted to Australian dollars in 2008 using health system deflators	Mandating more moderate use of salt in breads, margarines and cereals is easily the cost-effective strategy for primary prevention of CVD—produces biggest gains in population health: 80000 (60000 to 100000) DALYs averted, and cost saving.	To achieve best value for money in the primary prevention of CVD, the Australian government must take a tougher approach in mandating limits on salt in processed foods (bread, margarine and cereal),	DALYs averted based on literature, Assumed average population effect of the intervention is sustained with ongoing delivery of the interventions	Cost effective (based on cost-effectiveness threshold of $50,000/DALY)
Smith-Spangler et al (49)., USA, 2010	Population strategies to decrease sodium intake and the burden of cardiovascular disease: a cost-effectiveness analysis.	CUA Societal	US adults aged 40 to 85	Two interventions: 1. voluntary maximum sodium targets for processed foods; 2. tax on sodium used for food production	Timeframe of costs and consequences evaluated: lifetime. Discount rate: 3%	A Markov model was constructed with 4 health states: well, acute myocardial infarction, acute stroke, and history of MI or stroke.	Per-person cost of collaborating with industry = annual budget of the United Kingdom Food Standards Agency in 2008 divided by the number of adults aged 40 to 85 years.; this was converted to U.S. dollars using the World Bank purchasing power parity coefficient and assuming that 15% of the Food Standards Agency's budget was spent on the salt campaign. Costs of reformulation incurred by manufacturers excluded. Assumed negligible costs of administering the tax strategy. Tax revenue would subsidize the cost of lower-sodium foods for low-income persons.	Costs reported in 2008 US dollars. Base case estimate (best estimate) and range reported.	Collaboration with industry that decreases mean population sodium intake by 9.5% averts 513 885 strokes and 480 358 MIs over cohort's lifetime, increasing QALYs by 2.1 million and saving $32.1 billion in medical costs. A tax on sodium that decreases population sodium intake by 6% increases QALYs by 1.3 million and saves $22.4 billion over the same period.	Collaboration with industry achieves greater health benefits and lower costs than sodium tax (assumed products not reformulated as a result of a tax, and demand for salty products is relatively unresponsive to prices)	QALYs gained based on literature, assumed that the full effect of each strategy on sodium consumption would be achieved immediately and last a lifetime.	Cost saving
Rubinstein et al (50)., Argentina, 2010	Estimation of the burden of cardiovascular disease attributable to modifiable risk factors and cost-effectiveness analysis of preventative interventions to reduce this burden in Argentina.	CUA Health sector	Argentinian population over 35 years old	Program to lower salt in bread (one of 6 CVD interventions considered—involved government, consumer associations and bakers Comparator: current practice	Intervention duration: 5 years, Timeframe of costs and consequences evaluated: annual. Discount rate 3%	An epidemiological model was built incorporating prevalence and distribution of risk factors. A population level comparative risk assessment for seven modifiable cardiovascular risk factors were included in the model to assess their impact on major cardiovascular events.	Costs included program-level expenses associated with management of the interventions (i.e. administration, training and information, dissemination by multiple media sources)	All costs were calculated in Argentine pesos for 2007 then expressed in international dollars.	Lowering salt intake in the population through reducing salt in bread = cost saving—672.80 DALYs saved	Cost savings	DALYs averted, intervention effects were based on literature with delivery ongoing for the duration of the evaluation.	Cost Saving (cost-effectiveness based on interventions with an ICER that is less than three times GDP per capita as a “cost-effective” intervention and as “very cost-effective” if ICER is less than the GDP per capita)
Rubinstein et al (51)., Argentina, 2009	Generalized cost-effectiveness analysis of a package of interventions to reduce cardiovascular disease in Buenos Aires	CUA Government sector	Population of Buenos Aires	Program with government, consumer associations and bakers to reduce salt in bread (one of 8 intervention targeting CVD). Comparator: no intervention	Timeframe of costs and consequences evaluated: 10 years. Discount rate 3%	Used WHO-CHOICE methodology, and a standard multi-state modeling tool, PopMod.	Program level costs associated with management of program (administration, training, information dissemination), meetings with bakers (local data or expert opinion), biochemical analysis	All costs were reported in Argentine pesos for 2005	Lowering salt intake in the population through reducing salt in bread was found to be cost saving (ARS$151 per DALY averted)	Implementing this strategy to reduce salt intake would lead to both QALY gains and savings in economic resources.	DALYs averted, intervention effects were based on literature and assumed to be maintained.	Cost saving (cost-effectiveness based on a cost of below $1,000 per DALY).
Ha et al (52)., Vietnam, 2010	Cost-effectiveness analysis of interventions to prevent cardiovascular disease in Vietnam	CUA Societal	National Population	Health education through mass media education to reduce salt intake, and voluntary reduction in salt content of processed foods. Comparator: current practice	Timeframe of costs and consequences evaluated: 10 years. Discount rate: 3%	Used WHO-CHOICE methods and analytical models were employed, including PopMod model to estimate lifetime health gains	Mass media (local data on program management staff and different forms of media costs, posters), laboratory costs	Costs were measured in Vietnamese Dong for the year 2007	A mass media education program to reduce salt intake is the most cost effective (US$118/DALY averted)	A health education program to reduce salt intake and a combined mass media program on salt/tobacco/cholesterol are the best use of the government budget	DALYs averted, intervention effects were based on literature and assumed to be maintained.	Cost effective (based a cost-effectiveness ratio of less than three times GDP per capita).
Webb (53)., USA, 2014.	Population strategies to decrease sodium intake: A global cost-effectiveness analysis	CUA Government	Populations of 187 countries	A single combined 10 year intervention, based on UK's salt initiative, comprising legislation or voluntary regulation plus a public health campaign. Comparator: no intervention	Intervention duration: 10 years, Timeframe of costs and consequences evaluated: annual. Discount rate: 3%	Modelled analysis drawing on Global Burden of Disease data and effect sizes from previously published meta-analyses. Nation-specific impacts on mortality and disability-adjusted life years (DALYs) were modeled using comparative risk assessment, based on various scenarios including 10%, 30%, 1 g/d, and 3 g/d achieved sodium reductions over 10 yrs.	Nation-specific costs (based on WHO NCD Costing Tool. Cost categories were human resources (program management, promotion, media, advocacy, law enforcement, inspection, and national technical level assistance), training, meetings and mass media. Cost-effectiveness (CE) was evaluated as PPP-adjusted international $ per DALY saved over 10 yrs.	Costs reported in international dollars (local currency divided by country's US purchasing power parity). 2008 local currency costs converted to 2012 international dollars	A 10% sodium reduction within each country would avert average 5,655,000 CVD-related DALYs/year, at an average per capita cost of 1.11 international dollars over the 10 yr. intervention. The average CE ratio was I$207/DALY. Across 21 world regions, sodium reduction would be most cost effective in South Asia and East/Southeast Asia.	National education and industry-agreement strategies to reduce dietary sodium would have substantial impacts on CVD and be extremely cost-effective in nearly every country worldwide.	DALYs averted based on literature, the intervention efficacy is assumed to scale up linearly over the implementation period, having 10% of the full effect in the first year, 20% in the second, and so on, reaching full efficacy in the final year.	Cost effective (cost-effectiveness based on a cost-effectiveness ratio of less than three times GDP per capita).
Barton et al (54)., England and Wales, 2011	Effectiveness and cost effectiveness of cardiovascular disease prevention in whole populations: modelling study	CUA Healthcare	National populations of England and Wales	One salt intervention (of two interventions designed to target CVD)—legislation to reduce salt intake; current practice	Timeframe of costs and consequences evaluated: 10 years. Discount rate 3.5%	Generic spreadsheet model to quantify reduction in CVD over 10 years, assuming the benefits apply consistently for men and women across age and risk groups.	Intervention costs not specified. Estimated the expected lifetime costs, life years, and QALYs after a first cardiovascular event as a function of age and sex. These were compared with life expectancy without an event to determine the loss in life years and QALYs from such an event.	Costs are reported in British Pounds	Reducing salt intake by 3 g/day (conservative estimate of effect of intervention) might reduce mean population SBP by approximately 2.5 mm Hg. This would equate to a 2% decrease in the risk reduction model, and prevent 4450 deaths from CVD, with total discounted savings of approximately £347m over 10 years, representing equivalent annual savings of approximately £40m.	Any intervention that achieved even a modest population-wide reduction in any major cardiovascular risk factor would produce a net cost saving to the NHS, as well as improving health.	QALYs, savings in health care costs, cardiovascular events avoided, intervention effects are assumed to be maintained over the 10 years.	Cost saving
Ortegon et al (55)., Sub-Saharan Africa and South East Asia, 2012	Cost effectiveness of strategies to combat cardiovascular disease, diabetes, and tobacco use in sub-Saharan Africa and South East Asia: mathematical modelling study	CUA Healthcare perspective	Population of Sub-Saharan Africa and South East Asia	Voluntary or regulatory reduction in salt in processed foods (amongst 123 single or combined prevention and treatment strategies for CVD, diabetes and smoking) Comparator: current practice	Intervention timeframe: 10 years. Timeframe of costs and consequences evaluated: 100 years (lifetime). Discount rate: 3%	Follows WHO-CHOICE project methodology and builds on previous analyses of public health interventions to lower systolic blood pressure and cholesterol and of tobacco use.	Program costs including administration and planning, media and communications, training, evaluation and monitoring	Costs calculated for a 10 year period of implementation and expressed in international dollars for 2005.	Voluntary reduction—African sub-region $591/DALY averted; SE Asia $197 /DALY. Legislated reduction Africa $321/DALY, SE Asia $901,991	Salt reduction strategies feature amongst a set of population-wide and individual strategies for prevention and control of cardiovascular disease that are inexpensive and cost effective in low resource settings.	DALYs averted based on literature, intervention effects are assumed to be maintained over time	Cost saving (cost-effective interventions defined as if it produces a healthy year of life for less than three times the GDP per capita, and as “very cost effective” if it produces a healthy year of life for less than the GDP per capita).

**Table 3:** Summary of study characteristics.

Study Characteristics	Number of studies identified
**Study type**	
CEA	0
CUA	14
CBA	0
**Study perspectives**	
Societal	6
Health Sector or healthcare perspective	5
Government	2
Not specified	1
**Comparator selected**	
Current practice	12
No intervention	2
**Country or region income level**	
High income countries	10
Low and middle income countries or regions	4

#### Target settings and populations

The identified articles contained economic evaluations of interventions from a wide range of countries. Four were from low and middle income countries or regions [43,44,52,55], and 10 were from high income countries as classified by the World Bank [57]. The former group included studies from Vietnam, Syria and the Middle East (four countries in one study) and South-East Asia & Sub Saharan Africa. The latter group comprised four studies from the USA, England, Australia and Argentina, and Norway. The target group for the majority of studies was a national population; however one study targeted the population of a single city (Buenos Aries), whilst three were regional studies targeting multiple countries.

Seven of the 14 studies evaluated interventions which targeted the whole population of either a specific country [46,52], multiple countries [44,53,54], several regions [47,55], or a city [51]. Mason et al. [44] evaluated the intervention separately for the population of four Eastern Mediterranean countries (Palestine, Syria, Tunisia and Turkey), whilst Webb [53] modelled results separately for 187 different countries worldwide, Murray et al [47] for 14 epidemiological sub regions and Ortegon et al [55] for the populations of sub-Saharan Africa and South East Asia. Rubinstein 2009 focused on the city population of Buenos Aires [51]. Cobiac et al [46] and Ha et al [52] evaluated intervention for the populations of Australia and Vietnam respectively.

Of the 14 studies, the interventions were targeted at adults of varying age ranges. Four targeted young to middle age adults (35 or 40 years and over) [42,48–50], although the latter study was confined to adults (35–85 years) who had never experienced a CVD event. Two studies lowered the youngest age to between late adolescence or early adult years (between 16–25 years and upwards) [43,45]. There were no studies focusing exclusively on children.

#### Study perspective

The economic perspective of a study is important in determining the costs and benefits included. Six of the studies [42,44,45,47,49,52] purport to include a societal perspective, which means that all costs and benefits are included irrespective of who incurs them. The remaining studies reported from a health sector perspective [46,48,50,54,55], or government perspective [51,53]. The perspective taken by Wilcox et al. [43] was not specified.

#### Interventions and comparator

A range of interventions aiming to reduce sodium consumption were identified. These consisted of activities aiming to influence both the supply and demand side of the food system. Supply side interventions aimed to alter the available food by providing access to lower sodium options. The main example is product reformulation (both voluntary and mandatory) to reduce the salt content of food. Demand side interventions aimed to influence demand by changing people’s behavior so that they select lower sodium options. Examples included health promotion campaigns, labeling of salt content on packaged food and taxes on salty food products.

All interventions analysed were compared to either the status quo (current practice) or a null comparator. The latter, based on WHO-CHOICE methodology [58], entails an assumption of no interventions being in place, meaning that the intervention is compared to a situation of no costs and no interventions.

Over half of the studies (8/14) evaluated multiple sodium reduction interventions [43–47,49,52,55], while the remaining six studies evaluated only one sodium reduction strategy [42,48,50–54]. Seven of the studies [42–46,49,53] were focused exclusively on sodium reduction strategies, whilst in the other seven studies [47,48,50–52,54,55], a broader focus on the reduction of cardiovascular disease meant that the salt reduction interventions were evaluated along with a range of other non-salt initiatives. As an illustration of the latter, Murray et al 2013 [47] considered three salt interventions (health education through mass media, legislation and voluntary agreements on food labelling and salt content) amongst a total of 17 population and individual strategies to lower systolic blood pressure and cholesterol. Likewise, Cobiac et al. 2012 [48] included the mandatory reduction of salt in the manufacture of breads, margarines and cereals as part of a broader study of nine interventions exploring the best value for money in the primary prevention of cardiovascular disease. Of these eight studies, three evaluated two salt interventions [49,52,55], three had three interventions [43,44,46] and two had four interventions [45,47].

In the case of two of the six studies evaluating one salt reduction intervention only [42,53], the intervention was a multi-component intervention targeting sodium reduction, comprising product reformulation with a health promotion/education component. The other four were single component interventions [48,50,51,54].

The majority of the studies included a product reformulation strategy designed to reduce the sodium content of processed foods. Five studies [45–47,53,55] evaluated the cost-effectiveness of both voluntary and mandatory (regulatory) measures to restrict the salt content of processed foods, whilst four [43,44,48,54] included mandatory reformulation and five [42,49–52] included voluntary programs targeting the food industry. The other salt reduction initiative common to seven of the papers was a health promotion/education program [42–45,47,52,53]. Whilst the English study by Collins et al. [45] specified a particular health program (Change4Life), and Ha et al. [52] specified health education via a mass media campaign, generally little detail was provided on the nature of the program.

Only two studies evaluated tax legislation for salt reduction. Selmer et al. [42] included taxes on salty foods and subsidies on products with less salt as components within a multi-pronged salt reduction program. In contrast to point-of-sale tax measures, Smith-Spangler et al. [49] evaluated the impact of a tax imposed on sodium used in food production.

All of the studies assumed that the full effect of the intervention would be maintained over time. This assumption seems reasonable for interventions such as product reformulation and tax legislation however this may not always be realistic for health promotion and education programs. Three studies [42,46,53] assumed that effects would appear gradually from the onset of the intervention and increase to full effect. Only study [48] explicitly mentions that the effect of the interventions is only assumed if the delivery of interventions is ongoing.

#### Time horizons

Economic evaluations should specify time horizons, both for the provision of the intervention itself and for tracking the associated costs/cost offsets and consequences. The evaluated duration of intervention delivery should ideally reflect how the intervention would be applied in real life. There were a range of study time lines in the identified studies; the studies generally do not justify their choice of time frame for tracking costs and benefits. Half of the studies (7/14) had a 10 year study time line in which the costs and consequences of the interventions were evaluated [43–45,52–54,59]. One study [42] had a 25 year timeline and one estimated annual costs and benefits [50]. The remaining five studies measured results over 100 years or the lifetime of the target group [46–49,55]. In the small number of studies which actually specified the intervention duration, it ranged from five to 25 years.

#### Study designs and models employed

All of the 14 studies entailed a cost-utility analysis where the incremental cost-effectiveness ratios were reported as a ratio of costs against a measure of utility. Four studies [42–45] reported cost per life years gained, and two [49,54], costs per quality-adjusted life years (QALY) gained. All of the other studies measured costs per disability-adjusted life year (DALYs) saved.

The studies employed various forms of simulation modelling to examine the impact of the specified intervention on population health. Four studies based their analytic model on the WHO-CHOICE methodology [47,51,52,55]—reductions in population attributable risks of cardiovascular events resulting from an intervention were calculated, and then translated into changes in population health using the standard multi-state model, Pop Mod. Pop Mod estimates the lifetime health gains for each age and sex cohort of the given population (divided into different health states) both with and without the intervention. Three studies [43–45] used country specific versions of IMPACT, a comprehensive, validated coronary heart disease (CHD) model to estimate the reduction in CHD mortality stemming from an intervention. Other studies [42,48,49] developed purpose-built Markov models which assume that each participant is always in one of a finite number of discrete health states and events are represented as transitions from one state to another.

#### Discount rates

The majority of studies (11/14) applied a 3% discount rate to costs and benefits, whilst Collins et al [45] and Barton et al [54] used a 3.5% rate and Selmer et al [42] a 5% rate. The choice of discount rate was expected to vary between settings and location but most of the studies did not justify their choice of rate level.

#### Resource use costing

Items included in the cost measurement varied depending on the study purpose and perspective and the nature and number of intervention(s) being evaluated. Some studies such as Wilcox et al [43] and Collins et al [45], assumed a broad, societal approach to costing, in order to facilitate the inclusion of costs to all sectors, including the food industry (for example, the costs of product reformulation and relabeling). Others adopted a narrower focus and confined themselves, for instance, to costs to government [51,53] or the health care sector [54].

#### Quality assessment of the studies

The reporting quality of the 14 studies was assessed against 24 checkpoints and allocated a score of 1 for each point that was met in full (symbolized as √), a score of 0.5 for each point that was partially met (symbolized as ≠) and a score of 0 for each point that was not met (symbolized as X) (Table 4). The majority of studies (8/14) [44,46,48–52,55] were found to be of excellent reporting quality (scoring 85% or higher), with the remaining five to be of ‘very good’ quality (scoring 70–85%) [42,43,45,47,54] and one to be of ‘good’ quality (scoring 55–70%) [53].

**Table 4:** Quality Assessment Results against CHEERS Checklist.

	Title Identified as economic evaluation	Structured abstract	Intro provides context and a clear study question	Population characteristics	Setting and location	Study Perspective	Comparators described	Time horizon	Discount rate
**Author:**	1	2	3	4	5	6	7	8	9
Selmer et al 2010	X	√	√	√	√	≠	√	≠	X
Wilcox 2014	√	≠	√	√	√	X	√	≠	√
Mason et al 2014	√	√	√	√	√	≠	√	≠	√
Colins et al 2014	√	√	√	√	√	≠	√	≠	√
Cobiac et al 2010	√	√	√	√	√	√	√	√	√
Murray et al 2005	X	√	√	√	√	≠	√	≠	√
Cobiac et al 2012	√	≠	√	√	√	√	√	X	√
Smith-Spangler et al 2010	√	√	√	√	√	√	√	≠	≠
Rubinstein 2010	√	√	√	√	√	√	√	√	≠
Rubenstein 2009	√	√	√	√	√	√	√	≠	≠
Ha & Chisholm	√	√	√	√	√	√	√	√	√
Webb 2012	√	X	√	√	X	X	√	≠	≠
Barton et al 2011	√	√	√	√	√	X	√	≠	√
Ortegon et al 2012	√	√	√	√	√	√	√	√	√
	Outcomes and relevance	Measurement of effectiveness	Pref based outcomes	Costs (unit costs and methods) or Costs model based studies	Currency, date and conversion	Model choice described	Model assumptions	Analysis methods	Parameters of values
**Author:**	10	11	12	13	14	15	16	17	18
Selmer et al 2010	√	√	NA	√	√	≠	√	X	√
Wilcox 2014	√	√	NA	√	√	≠	√	√	≠
Mason et al 2014	≠	√	NA	√	√	≠	√	√	√
Colins et al 2014	≠	√	NA	√	X	≠	√	√	√
Cobiac et al 2010	≠	√	√	√	√	√	√	√	√
Murray et al 2005	√	√	X	√	√	≠	√	√	X
Cobiac et al 2012	√	√	√	√	√	≠	√	√	√
Smith-Spangler et al 2010	≠	√	√	√	√	≠	√	√	√
Rubinstein 2010	≠	√	√	√	√	≠	√	√	√
Rubenstein 2009	√	√	√	√	√	≠	√	√	√
Ha & Chisholm	√	√	√	√	√	√	√	√	√
Webb 2012	≠	√	√	√	√	≠	√	√	X
Barton et al 2011	≠	√	√	√	√	≠	√	√	X
Ortegon et al 2012	≠	√	√	√	√	√	√	√	√
	Incremental costs	Sensitivity of incremental costs or model sensitivity analyses	Heterogenity explained	Findings and limitations	Funding source	Potential conflict of interest			
**Author:**	19	20	21	22	23	24	**Total**		**%**
Selmer et al 2010	X	√	√	≠	≠	√	16.5/23		72%
Wilcox 2014	√	√	NA	√	≠	X	17.5/22		80%
Mason et al 2014	√	√	√	√	√	√	21/23		91%
Colins et al 2014	√	√	√	√	√	X	19/23		83%
Cobiac et al 2010	√	√	√	√	√	√	23.5/24		98%
Murray et al 2005	√	√	√	√	≠	√	19/24		79%
Cobiac et al 2012	√	√	√	√	√	√	22/24		92%
Smith-Spangler et al 2010	√	√	NA	√	√	√	21/23		91%
Rubinstein 2010	√	√	NA	√	≠	√	21/23		91%
Rubenstein 2009	√	√	NA	√	≠	√	21/23		91%
Ha & Chisholm	√	√	NA	√	≠	X	21.5/23		93%
Webb 2012	≠	√	√	√	X	X	15.5/24		65%
Barton et al 2011	≠	√	√	√	√	√	20/24		83%
Ortegon et al 2012	√	√	√	√	√	√	23.5/24		98%

The two criteria which were least well addressed were the time horizon and model choice. Whilst the time horizon for each study was generally specified, most studies omitted to provide reasons for choice. Likewise, very few studies provided justification for their choice of economic model. Other key areas where studies lost quality points related to study perspective (sometimes it was not explicitly stated or related to the costs included) and health outcomes (where their relevance was not made clear). It should be noted that the assessment of reporting quality is not indicative of the quality of the actual study results.

### Cost-effectiveness results

All of the fourteen studies concluded that their specified interventions targeting reductions in sodium consumption were cost-effective, and in the majority of cases, were cost saving (in other words, they resulted in health gains at a lower cost, measured against the comparator) (Table 5). For example, Barton et al. [54] concluded that any sodium reduction initiative that achieved even a modest population-wide reduction in any major cardiovascular risk factor would produce a net cost saving to United Kingdom’s National Health Service.

**Table 5:** Summary of cost-effectiveness results.

Intervention type	Number of studies evaluated	Number cost- effective	Number cost saving
Both voluntary and mandatory reformulation	5	3	2
Mandatory reformulation	4	1	3
Voluntary programs targeting the food industry	5	1	4
Health promotion/education programs	7	1	6
Tax legislation	2	0	2

Many studies examined the cost-effectiveness of a combination of interventions making it sometimes difficult to ascertain the effectiveness of a single intervention. In the seven studies [47,48,50–52,54,55] which evaluated both salt reduction strategies and other non-salt strategies to reduce poor cardiovascular outcomes, the strategies focused on salt reduction generally represented the best ‘value-for-money’ given their low-cost and population wide impacts. For example, Ha et al. [52] evaluated 12 population and individual level interventions to prevent cardiovascular disease in Vietnam and found a mass media campaign to reduce salt intake as the most cost-effective. Likewise, Cobiac et al 2012 [48] showed that mandating the more moderate use of salt in breads, margarines and cereals was easily the most cost-effective strategy for primary prevention of cardiovascular disease in Australia.

Whilst the results are generally not comparable between studies due to the heterogeneous nature of the methods used, the studies that evaluated multiple salt interventions indicate that some initiatives are consistently more cost-effective than others. Mandatory product reformulation was found to be substantially more cost-effective than the food industry sector undertaking voluntary reformulation [45–48]. The 2010 study by Cobiac et al. [46] found that making recommended limits for salt in bread, margarine and cereal products mandatory would potentially avert 18% of the disease burden arising from excessive salt consumption which was 20 times greater than the health gains achieved with the voluntary approach.

There was also evidence from two studies that multiple interventions working together are likely to be more cost-effective than any single intervention (e.g. [43,44]. Mason et al. [44] found that in all four Eastern Mediterranean countries targeted a comprehensive strategy of health education, food labelling and mandatory product reformulation would produce the greatest benefit in terms of life years gained and cost savings.

## Discussion

The economic evaluations of the identified studies indicate that interventions to reduce sodium consumption generally represent excellent value for money; or in other words, are either cost saving (more health gains at lower cost) or cost-effective (more health gains but at some additional cost). Most of the interventions are low cost in terms of their implementation costs, but produce significant long-term improvements in population health, thereby resulting in sizeable cost savings to society by substantially decreasing the cardiovascular disease burden and associated health care expenditure. Interventions focusing on curbing salt intake were shown to be more cost-effective in avoiding poor cardiovascular disease outcomes than other non-salt strategies. This finding was in line with results from previous studies [60–62].

Whilst most of the 14 studies were from high income countries, there were several studies in middle or low income countries. The majority of the studies have been published in the past ten years. Whilst growing attention is being given to effective interventions to reduce salt consumption, very few interventions to date have been subjected to economic evaluation. Also, the interventions which have been evaluated in terms of their economic credentials are narrow in terms of their content; most related to product reformulation, relabeling, or health promotion programs, with only a couple targeting tax legislation.

A recent systematic review of salt reduction initiatives around the world identified interventions in different categories: food reformulation, consumer education, front of pack labelling and interventions in public institution settings and taxation [23]. This review found that economic evaluations have been completed for all sodium reduction intervention categories except for the ‘interventions in public institution settings’ category. There was also one study identified [49] relating to sodium taxation. This indicates a gap in existing literature and a need for economic evaluations of these different interventions.

None of the identified studies were based on actual implementation and evaluation of interventions. Instead the interventions were simulated using economic modelling and intervention effectiveness data were drawn from external sources or the academic literature. None of the papers made explicit mention of procedures for checking their models. Five of the studies used an existing validated model for their analyses. Three of the studies [43–45] reported using the existing validated IMPACT CHD model to compare their results, whilst two of the studies [47,51] used PopMod to model their analyses. Future evidence would be strengthened by the actual implementation of intervention trials within real-life settings. Despite this reliance on modelling and associated assumptions, the studies evaluated are important as model-based health economic evaluations are today widely accepted as policy-making tools that can inform resource allocation decisions.

A key strength of this review is the systematic and comprehensive method of data collection. A comprehensive search strategy was employed encompassing both peer reviewed and grey literature. The quality assessment of the economic evaluations undertaken as part of this review adds strength to the conclusions since all studies were found to be of good, very good or excellent reporting quality. The results in this review are limited to those published in English representing a potential limitation. Another limitation is that studies identified were not based on actual implementation of intervention(s) and the associated collection of new empirical data. Given the result of the studies are based on modelling and assumed costs and effectiveness, researcher bias may have influenced these findings. All studies identifies were based on modelling where inputs were drawn from published datasets, existing literature or expert advice. As the results from the study rely heavily on modelling, there is a need for the effectiveness of new interventions to be evaluated in the field using strong study designs and parallel economic evaluations.

## Conclusions

Reducing the consumption of salt is now seen as a key priority in many strategies targeting the prevention of cardiovascular disease but relatively few interventions designed to lower salt intake have been rigorously evaluated. Even fewer have been examined in terms of their economic credentials. Nevertheless, the economic evaluations identified in this field suggest that salt lowering strategies are potentially cost effective and offer better value-for-money than many other non-salt strategies. In addition to simulation modelling studies, there is an urgent need for the effectiveness of salt interventions to be actually evaluated in the field using strong study designs, and economic evaluations conducted in parallel.

## Supporting information

S1 File PRISMA 2009 checklist. (DOC)
